# Icariin ameliorates IgA nephropathy by inhibition of nuclear factor kappa b/Nlrp3 pathway

**DOI:** 10.1002/2211-5463.12161

**Published:** 2016-12-20

**Authors:** Lei Zhang, Xing‐Zhi Wang, Yu‐Shu Li, Lei Zhang, Li‐Rong Hao

**Affiliations:** ^1^ 2nd Department of NephrologyThe 1st Affiliated Hospital of Harbin Medical UniversityHeilongjiang ProvinceChina

**Keywords:** icariin, IgA nephropathy, inflammation, Nlrp3 inflammasome, nuclear factor kappa b

## Abstract

Immunoglobulin A nephropathy (IgAN) is the most frequent form of glomerulonephritis, which is characterized by glomerular proliferation and renal inflammation. Icariin is a flavonoid from the Chinese herb Epimedium, and its anti‐inflammatory effect has been reported. This study aimed to investigate the effects of icariin on the renal damage in IgAN rats and the mechanisms behind these effects. IgAN model was established in Sprague–Dawley rats by oral and intravenous immunization with bovine gamma‐globulin for 12 weeks, and rats were treated with icariin from 12 to 18 weeks. At the end of experimental period, kidneys, urine, and blood samples were collected for further analysis. Our results showed that icariin ameliorated the increase in the levels of proteinuria, serum creatinine, and urea nitrogen without severe side effects. IgAN rats exhibited significantly increased IgA deposition, mesangial matrix expansion, and glomerular fibrosis, while icariin treatment markedly attenuated these alterations. Moreover, treatment with icariin also dramatically blocked nuclear factor kappa b (NF‐κB) nuclear translocation and Nlrp3 inflammasome activation in IgAN rats, leading to reduced downstream proinflammatory cytokines production. Mechanistically, we found that icariin treatment inhibited IKKβ and IκBα phosphorylation and IκBα degradation in IgAN rats. Our data demonstrate that icariin ameliorates renal damage in IgAN rats via inhibition of NF‐κB‐mediated Nlrp3 inflammasome activation. These findings provide insight into an application of icariin for the treatment of IgAN disease, and represent a novel mechanism behind these effects.

**Abbreviations**
ASC: apoptosis‐associated speck‐like protein containing a CARD
Col‐IV: collagen IV
IKKβ: IκB kinase complex β
IκBα: inhibitors of κBα
NF‐κB: nuclear factor kappa b
NLRP3: nacht domain‐, leucine‐rich repeat‐, and pyrin domain‐containing protein 3
TGF‐β: transforming growth factor‐β

Immunoglobulin A nephropathy (IgAN) is one of the most common forms of glomerulonephritis and the leading cause of end‐stage renal disease (ESRD) in the world 1. Approximately 25–30% of IgAN patients require renal replacement therapy within 20–25 years, and an additional 20–40% develops to ESRD within 20 years of disease onset 2, 3. The costs of kidney transplantation and dialysis have become a huge financial burden to patients and society 4, 5. Therefore, it is of foremost importance to develop efficient agents for the treatment of IgAN.

Currently, IgAN is characterized by the deposition of IgA and by mesangial matrix expansion 6. However, little is known about the molecular pathway involved in these pathological abnormalities during IgAN progression. Recent studies have indicated that immune‐sensing inflammation with nuclear factor kappa b (NF‐κB) activation is considered as a critical step in IgAN prior to the development of ESRD in IgAN patients and animal model 7, 8, 9, 10, 11. Nlrp3 is a cytoplasmic multiprotein complex termed inflammasome, which comprises the ASC adaptor and triggers the activation of caspase‐1 and the maturation of proinflammatory cytokines interleukin (IL)‐18 and IL‐1β to execute inflammatory response 12, 13. Notably, previous studies have demonstrated that the levels of IL‐18 and IL‐1β, which are two key markers of Nlrp3 inflammasome activation, were elevated in serum and urine of IgAN patients 8, 9, suggesting Nlrp3 inflammasome may play an important role in IgAN by regulating inflammation.

Icariin, a major constituent of flavonoids isolated from plants of genus Epimedium, has been shown effectiveness in anticancer, cardiovascular protection, and bone formation promotion, as well as anti‐inflammation function 14, 15. Previous studies have reported that icariin suppressed inflammatory response by attenuating various intracellular signaling pathways including Nlrp3 and NF‐κB signaling 15, 16. Furthermore, recent work showed that icariin could ameliorate the cisplatin‐induced nephrotoxicity via reducing NF‐κB activation and inflammation 15. In addition, icariin also effectively alleviated chronic renal failure induced by nephrectomy and diabetic nephropathy in rats 17, 18, 19. Thus, icariin may be a comprehensive protective agent for the treatment of renal diseases and their complications. However, the role of icariin in IgAN has not been studied so far. The aim of this study was to investigate the effect of icariin on renal function and morphological alternations in IgA rats, and explore the underlying mechanisms. Our findings may provide a novel application of icariin on IgAN, and lead to the development of a viable option for the treatment of IgAN.

## Materials and methods

### Materials and reagents

Icariin (purity ≥ 98%) was obtained from the National Institute for the Control of Pharmaceutical and Biological Products (Beijing, China) and dissolved in saline with ultrasonication. Unless otherwise indicated, all chemicals were purchased from Sigma‐Aldrich (St. Louis, MO, USA).

### Animal model and experimental design

Six‐week‐old male Sprague–Dawley rats (180–200 g) were obtained from the Experimental Animal Center of Harbin Medical University (Harbin, China). All animals were maintained at controlled temperature (22–25 °C) under a 12‐h light/12‐h dark cycle, with free access to food and water. The experimental protocols for animals were approved by Institutional Animal Ethical Committee of Harbin University and were performed in accordance with the ‘Guide for the Care and Use of Laboratory Animals’ of the National Institutes of Health in China.

A total of 60 rats were randomly distributed into four groups with 15 rats in each group: control, icariin, IgAN, and IgAN treated with icariin. The IgAN model was generated by continuous oral administration of 0.1% bovine gamma‐globulin (BGG) with 6 mm HCl in tap water for 8 weeks followed by an intravenous tail injection of 1 mg BGG daily for three successive days 20, 21, 22. For the control group, rats received saline instead of BGG. After the establishment of the IgAN model at the end of week 12, rats were administered with icariin (10 mg·kg^−1^·day^−1^) by gavage from 12 to 18 weeks. At the end of the experiment, the rats were weighed and anesthetized. Blood samples were collected and the serum was obtained after centrifugation at 2000 ***g*** for 5 min at 4 °C. Urine was collected for 24 h using metabolic cages. Kidneys were perfused with saline through the left ventricle and processed for pathologic evaluation, quantitative PCR, and western blot.

### Biochemical parameters analysis

The 24‐h urinary protein levels were measured using Coomassie Brilliant Blue assay. The levels of serum creatinine, urea nitrogen, and total protein were examined by available commercial kits (Hayward, CA, USA) according to manufacturer's instruction. Albumin, alanine aminotransferase (ALT), and aspartate aminotransferase (AST) levels were determined with an autoanalyzer (Beckman Instruments, Fullerton, CA, USA).

### Pathologic evaluation

Formalin‐fixed kidneys were embedded using optimal cutting temperature compound (OTC, Sakura, Japan) and 4‐μm sections were prepared. To evaluate IgA deposition in the glomeruli, FITC‐conjugated rabbit anti‐(rat IgA) antibody (dilution 1 : 100; Bethyl Laboratories, Montgomery, TX, USA) was employed for direct immunofluorescence as previously described 22. IgA deposition was observed by a laser confocal microscopy (Carl Zeiss, Oberkochen, Germany). To examine glomerular proliferation, sections were stained with hematoxylin‐eosin (HE) and periodic acid‐Schiff (PAS), respectively 22. The PAS‐positive area (mesangial area) in glomeruli was determined using quantitative image analysis software (pax‐it; Paxcam, Villa Park, IL, USA). The relative degree of cellular proliferation was evaluated by rating the PAS‐positive area occupying the area of glomeruli. A total of 20–30 glomeruli/rat were examined and the average percentage of glomerular matrix expansion was calculated for each rat. Masson trichrome staining was utilized to evaluate tubulointerstitial injuries and fibrosis as described previously 22. The extent of the increase in interstitial fibrotic area was determined with quantitative image analysis software by rating the area occupied by Masson trichrome‐positive area as previously described 23. For immunohistochemical staining, sections were incubated with primary antibodies against transforming growth factor‐β1 (TGF‐β1), Fibronectin1, Nlrp3, ASC, Caspase‐1, IL‐18 (Santa Cruz, CA, USA), collagen IV (Col‐IV) (Southern Biotech, Birmingham, AL, USA) and IL‐1β (Cell Signaling Technology, Beverly, MA, USA) at a 1 : 100 dilution overnight at 4 °C. Signals were detected with appropriate biotinylated secondary antibodies and then developed with DAB chromogen (Vector Laboratories, Peterborough, UK). All images were acquired using a light microscopy (Olympus, Tokyo, Japan). The signal intensity of TGF‐β1, Col‐IV, Fibronectin1, Nlrp3, ASC, Caspase‐1, IL‐18, and IL‐1β was quantitated in 20–30 glomeruli/rat as the positive area expressed as a percentage of the area using pax‐it software.

### Quantitative PCR

Total RNA was extracted from the renal cortical tissues using TRIzol reagent (Takara, Dalian, China) and RNA concentrations were determined by UV spectrometry. One microgram of RNA was reverse‐transcribed using High Capacity cDNA Archive Kit (Applied Biosystems, Foster City, CA, USA), and quantitative PCR was performed with ABI 7500 Real‐Time PCR System (Applied Biosystems) using SYBR Premix Ex Taq (Takara). The primer sequences for rat TGF‐β1, Col‐IV, Fibronectin1, Nlrp3, ASC, Caspase‐1, IL‐18, IL‐1β, and GAPDH are listed in Table 1. Amplifications were carried out in 20‐μL reactions under the following cycling conditions: 95 °C for 5 min followed by 40 cycles of denaturation (95 °C for 10 s), annealing (60 °C for 30 s), and extension (72 °C for 1 min). The expression for each gene was calculated using the 2^−ΔΔCT^ method and normalized against the internal control GAPDH.

**Table 1:** The primer sequences used for quantitative PCR

Genes	Primer sequences (5′–3′)	
*TGF‐*β*1*	Forward	GCCTGAGTGGCTGTCTTTTGA
	Reverse	GAAGCGAAAGCCCTGTATTCC
*Col‐IV*	Forward	GGGGTCGGGCTGGGAGTGAT
	Reverse	GCTGGCCGTCCATACCCGTG
*Fibronectin 1*	Forward	CCAAGGTCAATCCACACCCC
	Reverse	AGAACAGGGCAACCAACTGT
*Nlrp3*	Forward	AGCTGCTCTTTGAGCCTGAG
	Reverse	TCTGCTAGGCTCTTTGGTGC
*ASC*	Forward	ACAGTACCAGGCAGTTCGTG
	Reverse	GGTCTGTCACCAAGTAGGGC
*Caspase‐1*	Forward	GACCGAGTGGTTCCCTCAAG
	Reverse	GACGTGTACGAGTGGGTGTT
*IL‐18*	Forward	AACCGCAGTAATACGGAGCA
	Reverse	TCTGGGATTCGTTGGCTGTT
*IL‐1*β	Forward	CCTTGTCGAATGGGCAGT
	Reverse	CAGGGAGGGAAACACACGTT
*GAPDH*	Forward	GGCATTGCTCTCAATGACAA
	Reverse	TGTGAGGGAGATGCTCAGTG

### Western blot

Total protein was isolated from renal cortical tissues in protein lysis buffer (Beyotime, Jiangsu, China). Cytoplasmic and nuclear proteins were extracted from renal cortical tissues using a Nuclear/Cytosol Fractionation Kit (BioVision, Milpitas, CA, USA) according to the manufacturer's instructions. The protein concentrations were determined using a bicinchoninic acid assay kit (Beyotime). Protein from each sample (50 μg) was separated by 10% SDS‐acrylamide gel and then transferred onto PVDF membranes (Millipore, Bedford, MA, USA). After blocking with 5% nonfat milk, the membranes were incubated with the following primary antibodies at 4 °C overnight: p65, p50, p‐IκBα, IκBα, and p‐IKKβ (dilution 1 : 1000; Cell Signaling Technology); Lamin B and β‐actin (diluted 1 : 2000) (Santa Cruz). On the second day, the membranes were washed and incubated with appropriate horseradish peroxidase‐conjugated secondary antibodies (anti‐rabbit or anti goat; Santa Cruz) at a 1 : 4000 dilution. Blots were visualized with enhanced chemiluminescence (Thermo, Rockford, IL, USA) and the relative density was quantitated using the image‐pro plus software (Media Cybernetics, Rockville, MD, USA).

### Immunoprecipitation

Protein A/G agarose beads (Santa Cruz) was added to the protein lysate of renal cortical tissues to preclear nonspecific binding. Equal amounts of proteins were coincubated with inhibitors of κBα (IκBα) antibody and protein A/G agarose beads at 4 °C overnight. On the second day, the immunoprecipitates were washed with protein lysis buffer and then analyzed by western blot using ubiquitin antibody (dilution 1 : 1000; Cell Signaling Technology).

### Statistical analysis

The results were expressed as mean value ± standard error of mean (SEM). Comparisons among groups and two groups were analyzed by one‐way ANOVA with Bonferroni correction and Mann–Whitney *U*‐test, respectively. Statistical analysis was conducted using spss 17.0 statistical software (SPSS Inc., Chicago, IL, USA). *P* < 0.05 was considered statistically significant.

## Results

### Effect of icariin on functional parameters in IgAN model

Renal function was analyzed at the end of the 18‐week experimental period (Table 2). The rats with IgA nephropathy showed a marked increase in proteinuria when compared with the control group. In contrast, icariin treatment significantly decreased proteinuria level in IgAN rats. Moreover, IgAN was also associated with slightly increased levels of serum creatinine and blood urea nitrogen, which were suppressed by icariin treatment. However, body weight and the levels of total protein and albumin remained throughout the experimental period. Of note, no evidence of renal function damage was observed between control and control + icariin group. To further investigate the adverse effect of icariin treatment, we analyzed the liver function damage before and after icariin treatment. Although the proteinuria level was significantly increased in IgAN rats, plasma ALT and AST levels did not change among the groups, indicating icariin produces no liver toxicities. In addition, all animals grew well and no significant hair loss was observed prior to killing.

**Table 2:** Physiological characteristic of rats at the end of 18 weeks. ALT, alanine aminotransferase; AST, aspartate aminotransferase

Parameter	Control	Control + Icariin	IgAN	IgAN + Icariin
Body weight (g)	482.4 ± 14.6	475.5 ± 16.7	470.2 ± 12.4	478.9 ± 10.9
Proteinuria (mg/24 h)	3.50 ± 0.51	3.33 ± 0.46	58.72 ± 8.90**	33.65 ± 5.29^##^
Serum creatinine (mg/dL)	0.69 ± 0.07	0.70 ± 0.09	0.90 ± 0.07*	0.75 ± 0.06^##^
Urea nitrogen (mg/dL)	16.68 ± 1.89	17.24 ± 2.23	23.75 ± 2.17*	20.88 ± 1.93^#^
Total protein (g/L)	62.64 ± 5.93	60.70 ± 5.27	65.95 ± 6.14	59.55 ± 6.92
Albumin (g/L)	14.01 ± 1.68	14.97 ± 1.87	13.74 ± 2.14	14.09 ± 1.57
ALT (U/L)	43.57 ± 5.87	44.68 ± 4.97	47.8 ± 5.04	46.81 ± 5.48
AST (U/L)	89.87 ± 7.14	92.47 ± 7.01	94.7 ± 5.47	92.87 ± 5.97

Values are mean ± SEM, *n* = 8–10 in each group

*P < 0.05, **P < 0.01 vs. control.

^**#**^ *P* < 0.01, ^**##**^ *P* < 0.01 vs. IgAN.

### Icariin inhibited IgA deposition and pathomorphological changes in glomeruli

**Figure 1:**
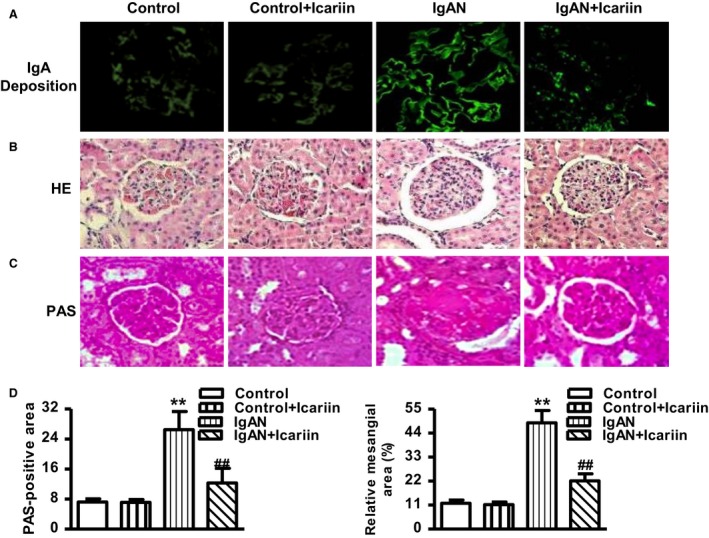
Effects of icariin renal pathology on the glomeruli of IgAN rats. Icariin was administrated daily by gavage to normal (Control) or IgAN rats. (A) IgA deposition in the glomeruli was visualized by direct immunofluorescence. (B,C) Representative images of hematoxylin and eosin (HE) (B) and periodic acid‐Schiff (PAS) (C) immunohistochemically stained rat glomeruli. (D and E) Quantitative assessment of PAS‐positive area (D) and the ratio of the mesangial matrix/glomerular area (E). The data were expressed as mean value ± SEM. **P < 0.01 vs. control; ^##^ *P* < 0.01 vs. IgAN,* n* = 8 in each group.

As shown in Fig. 1A, little IgA deposition was observed in control rats before or after icariin treatment. IgAN rats revealed increased IgA deposition at the end of experimental period, whereas icariin treatment significantly reduced the IgA deposition. We also observed the glomerular morphological changes by HE and PAS staining. The results of HE staining revealed that thickening of tubular basement membrane and hyperplasia of mesangial were obvious in IgAN group. After administration of icariin, the mesangial region showed no hyperplasia. In addition, the diameter of glomeruli was longer in IgAN group compared to control group, and this effect was dramatically inhibited after icariin treatment (Fig. 1B). Moreover, PAS staining also showed widening of the mesangial region and increased mesangial matrix in IgAN rats. In IgAN rats treated with icariin, obvious reduction in glomerular matrix protein was observed (Fig. 1C–E). The results indicate that icariin can attenuate these pathological abnormalities in IgAN rats.

### Icariin alleviated glomerular fibrosis in IgAN rats

**Figure 2:**
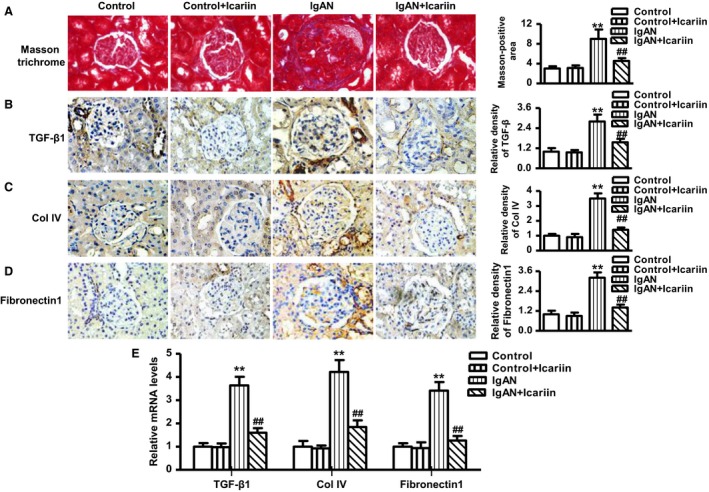
Effects of icariin on markers of glomerular fibrosis in IgAN rats. (A–D) Representative images of the glomeruli stained with Masson trichrome (A), TGF‐β1 (B), Col‐IV (C), and Fibronectin1 (D). (E) The mRNA expression of TGF‐β1, Col‐IV, and Fibronectin1 were measured by quantitative PCR. The data were expressed as mean value ± SEM. **P < 0.01 vs. control; ^##^ *P* < 0.01 vs. IgAN,* n* = 6–8 in each group.

Masson trichrome staining showed increased deposition of collagen fibrils in glomeruli in IgAN rats. However, icariin treatment diminished the deposition of collagen fibrils (Fig. 2A). Furthermore, we immunohistochemically stained the glomeruli using antibodies against TGF‐β1, Col‐IV, and Fibronectin1 as markers to estimate fibrosis development. Immunohistochemistry demonstrated that the protein expression of these profibrotic markers were significantly increased in the glomeruli of IgAN rats compared with control group, whereas administration of icariin was associated with reduced protein expression of the above markers (Fig. 2B–D). Consistent with the above results, the mRNA expression of TGF‐β1, Col‐IV, and Fibronectin1 were upregulated in rats with IgAN, which was attenuated by icariin treatment (Fig. 2E).

### Icariin suppressed Nlrp3 inflammasome activation and proinflammatory cytokine production in IgAN rat model

**Figure 3:**
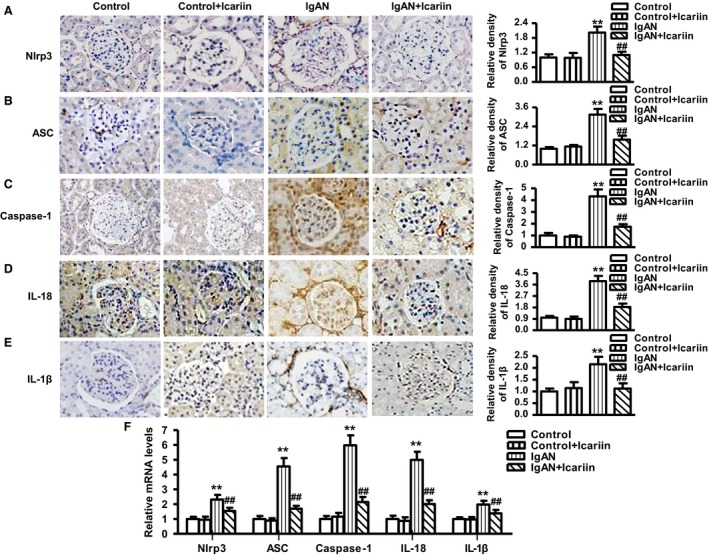
Effects of icariin on Nlrp3 inflammasome activation and proinflammatory cytokine production in IgAN rats. (A) Glomerular expression of Nlrp3 (A), ASC (B), Caspase‐1 (C), IL‐18 (D), and IL‐1β (E) as shown by immunohistochemical staining. (F) The mRNA expression of the above markers were determined by quantitative PCR. The results were expressed as mean value ± SEM. **P < 0.01 vs. control; ^##^ *P* < 0.01 vs. IgAN,* n* = 6–8 in each group.

To investigate whether the protective effect of icariin in IgAN rats was associated with the inhibition of inflammation, we measured Nlrp3 inflammasome activation and downstream proinflammatory cytokine production in glomeruli. As shown in Fig. 3A, the Nlrp3‐positive area was far more extensive in IgAN rats than in control rats. Icariin administration notably decreased the Nlrp3‐positive area in IgAN rats treated with icariin. As an indication of Nlrp3 activation, immunohistochemistry also revealed elevated protein expression of ASC, Caspase‐1, IL‐18, and IL‐1β in glomeruli, whereas icariin treatment significantly decreased the protein expression of the above proinflammatory cytokines (Fig. 3B–E). To further confirm our findings, we measured the mRNA expression of Nlrp3, ASC, Caspase‐1, IL‐18, and IL‐1β, which were consistent with the above results (Fig. 3F).

### Icariin attenuates NF‐κB activation in IgAN model rats

**Figure 4:**
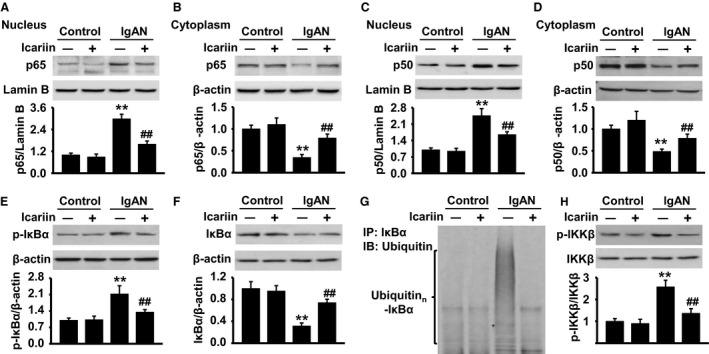
Icariin attenuates NF‐κB activation via inhibition of IKKβ/IκBα pathway. Control or IgAN rats were treated with or without icariin by gavage for 6 weeks. (A–D) Nuclear and cytoplasmic fractions of renal cortical tissues were isolated and NF‐κB nuclear translocation was analyzed by western blot using p65 (A,B) and p50 (C,D) antibodies. (E and F) IκBα phosphorylation (E) and protein expression (F) were determined by western blot. (G) Protein lysate form renal cortical tissues were immunoprecipitated with IκBα antibody and the immunoprecipitates were blotted with ubiquitin antibody to reveal ubiquitination of IκBα. (H) IKKβ phosphorylation and total protein expression were analyzed by western blot. The results were expressed as mean value ± SEM. **P < 0.01 vs. control; ^##^ *P* < 0.01 vs. IgAN,* n* = 6 in each group.

We next investigated the underlying mechanisms that might be involved in the protective effect of icariin. Since activation of NF‐κB pathway is indicated in the acceleration and progression of IgAN 24, NF‐κB nuclear accumulation was analyzed in renal cortical tissues by western blotting using p65 and p50 antibodies. In control rats treated with or without icariin, the majority of p65 and p50 remained in cytoplasmic fractions. However, IgAN rats showed significantly enhanced nuclear accumulation of p65 and p50, and this effect was markedly blocked by icariin treatment (Fig. 4A–D). To further explore how icariin inhibits NF‐κB activation, we determined the effect of icariin on NF‐κB upstream signaling pathway IκB kinase complex β (IKKβ)/IκBα. As shown in Fig. 4E, phosphorylation of IkBα was dramatically induced in IgAN rats, whereas icariin treatment was associated with inhibited the phosphorylation of IkBα. Moreover, IgAN rats revealed notably reduced IkBα protein expression. Nevertheless, icariin treatment remarkably antagonized against IkBα protein degradation in IgAN rats (Fig. 4F). The result was further confirmed by immunoprecipitation analysis, as evidenced that icariin treatment significantly attenuated the elevation of IκBα ubiquitination in IgAN rats (Fig. 4G). In addition, administration of icariin obviously inhibited IKKβ phosphorylation, as compared with those from IgAN rats (Fig. 4H). These findings indicate that icariin attenuates NF‐κB activation via inhibition of IkBα degradation and IkBα and IKKβ phosphorylation.

## Discussion

The present study showed that icariin treatment effectively ameliorated renal dysfunction and glomerular proliferation and fibrosis that are indicative of the progressive stage of IgAN in the rat model. To investigate the underlying mechanism, the protective effect of icariin was associated with reduced activation of NF‐κB/Nlrp3 pathway.

We firstly determined whether icariin is beneficial for the treatment of IgAN. The proteinuria levels and functional parameters in IgAN rats were analyzed after icariin treatment for 6 weeks. We found that the proteinuria level was dramatically increased, and the levels of serum creatinine and urea nitrogen were slightly higher in IgAN rats than those in control rats. However, icariin markedly ameliorated the increase in the levels of these above parameters. Although the animals showed heavy proteinuria accompanied by slightly high levels of serum creatinine and urea nitrogen in our IgAN model, the body weight and liver function of animals still remained stable at the end of the experimental period. This was consistent with clinical symptoms in IgAN patients 25. Moreover, the control rats treated with icariin revealed no evidence of kidney and liver functional damage during the entire study period, indicating the safety and nontoxicity for icariin treatment.

Clinically, abnormal renal functional parameters are highly associated with structural injury in IgAN disease, especially with IgA deposition and glomerular matrix expansion as well as renal fibrosis 26. Consistent with improvements in renal function, icariin treatment also reduced IgA deposition and mesangial matrix expansion in glomeruli of IgAN rats. Moreover, IgAN induced a marked elevation of profibrotic markers expression that was significantly inhibited by icariin treatment. These results suggest that icariin prevents the renal damage in glomeruli of IgAN rats by improving renal function and structure.

Multiple studies been reported that inflammation plays a pathogenic role in the evolution of IgAN 7, 9, 26. A clinical study in Finland enrolled total of 174 patients with IgAN found that there was a marked increase in inflammatory markers such s‐albumin, high‐sensitivity C‐reactive protein, and white blood cells, which were suggested to be associated with the progression of IgAN 7. Moreover, increasing clinical evidences further demonstrated that the production of proinflammatory cytokines such IL‐18 and IL‐1β were dramatically upregulated in IgAN patients and this is considered as a prognostic factor in these patients 8, 9, 10. It has been documented that mature IL‐18 and IL‐1β are generated via the activation of Nlrp3 inflammasome 13, 27. Initially, NF‐κB acts as a primary signaling that induces transcription and translation of pro‐IL‐18 and pro‐IL‐1β and activation of Nlrp3 inflammasome 12, 28. Then the latter further activates caspase‐1, which in turn cleaves mature IL‐18 and IL‐1β 27. A previous work in unilateral ureteral obstruction mice showed that the inflammation and renal fibrosis were associated with Nlrp3 inflammasome activation and the downstream proinflammatory cytokines production 29. In addition, an *in vitro* study also confirmed that IgA immune complex was mediated by the activation of Nlrp3 inflammasome 30. The above findings indicate that Nlrp3 inflammasome plays a role in various renal diseases including IgAN. In the present study, we found that Nlrp3 and downstream cytokines ASC, caspase‐1, IL‐18, and IL‐1β were all upregulated in the renal cortical tissues of IgA rats, whereas icariin treatment abrogated the levels of the above cytokines. Our data suggest that the inhibition of Nlrp3 inflammasome activation may be responsible for the protective effects of icariin on renal injury in IgAN rats.

Since NF‐κB is an important mediator of inflammatory disorder and induces Nlrp3 activation 24, 28, we next explored the possibility that whether icariin inhibited Nlrp3 inflammasome activation by regulating NF‐κB signaling. In resting state, NF‐κB containing p65 and p50 subunits resides in the cytoplasm with low transcriptional activity in an inactive complex with IκB proteins 31. Upon a variety of proinflammatory stimuli, the inhibitor of NF‐κB, IKKβ, is phosphorylated and this promotes IκBα phosphorylation at N‐terminal sites, which leads to the triggering of IκBα ubiquitin‐dependent degradation 32, 33. Consequently, p65 and p50 translocate from the cytoplasm to the nucleus, which promotes the downstream gene transcription and expression 24. In this study, our data demonstrated that icariin treatment suppressed nuclear translocation of p65 and p50, blocked IKKβ and IκBα phosphorylation, and attenuated IκBα ubiquitin and degradation in the renal cortical tissues isolated from IgA rats. These results indicate inhibition of IKKβ and IκBα phosphorylation and IκBα ubiquitin may underly, at least partially, the inactivation of NF‐κB by icariin treatment.

## Conclusions

In summary, our data clearly demonstrate that icariin treatment ameliorates renal damage in IgAN rats, apparently by the inhibition of NF‐κB‐mediated Nlrp3 inflammasome activation. These findings suggest that icariin may be a potential therapeutic agent to prevent the onset and progression of IgAN.

## Author contributions

LH conceived and designed the experiments and contributed to the writing of the manuscript; LZ and XW performed the experiments; YL and LZ analyzed the data; LZ and LH designed the software used in analysis.
